# 3D-QSAR Studies of 1,2,4-Oxadiazole Derivatives as Sortase A Inhibitors

**DOI:** 10.1155/2021/6380336

**Published:** 2021-12-06

**Authors:** Neda Shakour, Farzin Hadizadeh, Prashant Kesharwani, Amirhossein Sahebkar

**Affiliations:** ^1^Department of Medicinal Chemistry, School of Pharmacy, Mashhad University of Medical Sciences, Mashhad, Iran; ^2^Student Research Committee, Faculty of Medicine, Mashhad University of Medical Sciences, Mashhad, Iran; ^3^Biotechnology Research Center, Pharmaceutical Technology Institute, Mashhad University of Medical Sciences, Mashhad, Iran; ^4^Department of Pharmaceutics, School of Pharmaceutical Education and Research, Jamia Hamdard, New Delhi 110062, India; ^5^Applied Biomedical Research Center, Mashhad University of Medical Sciences, Mashhad, Iran; ^6^Department of Biotechnology, School of Pharmacy, Mashhad University of Medical Sciences, Mashhad, Iran

## Abstract

Sortase A (SrtA) is an enzyme that catalyzes the attachment of proteins to the cell wall of Gram-positive bacterial membrane, preventing the spread of pathogenic bacterial strains. Here, one class of oxadiazole compounds was distinguished as an efficient inhibitor of SrtA via the “*S. aureus* Sortase A” substrate-based virtual screening. The current study on 3D-QSAR was done by utilizing preparation of the structure in the Schrödinger software suite and an assessment of 120 derivatives with the crystal structure of 1,2,4-oxadiazole which was extracted from the PDB data bank. The docking operation of the best compound in terms of pMIC (pMIC = 2.77) was done to determine the drug likeliness and binding form of 1,2,4-oxadiazole derivatives as antibiotics in the active site. Using the kNN-MFA way, seven models of 3D-QSAR were created and amongst them, and one model was selected as the best. The chosen model based on *q*^2^ (pred_*r*^2^) and *R*^2^ values related to the sixth factor of PLS illustrates better and more acceptable external and internal predictions. Values of crossvalidation (pred_*r*^2^), validation (*q*^2^), and *F* were observed 0.5479, 0.6319, and 179.0, respectively, for a test group including 24 molecules and the training group including 96 molecules. The external reliability outcomes showed that the acceptable and the selective 3D-QSAR model had a high predictive potential (*R*^2^ = 0.9235) which was confirmed by the *Y*-randomization test. Besides, the model applicability domain was described successfully to validate the estimation of the model.

## 1. Introduction

Sortase A (SrtA) is a polypeptide containing 206 amino acids. This enzyme speeds up two consecutive reactions: (a) transpeptidation and (b) thioesterification. SrtA is involved in the bacterial adhesion process and acts by attaching proteins holding LPXTG to lipid II [1–11]. SrtA inhibitors do not influence bacterial growth, but instead, they prevent the emergence of the virulence of pathogenic bacterial strains, thereby hindering infections produced by Staphylococcus aureus (*S. aureus*) or other bacteria of Gram-positive strain. To the surface membrane protein of *S. aureus*, sortase is attached which links it to the cell wall via transpeptidation [6, 10, 12], and needing a C-terminal regulates signal through a protected LPXTG motif [13–15]. *S. aureus* mutations with a deficiency of the srtA gene cannot display and bind some surface proteins which results in a disorder/disease such as animal infection [13, 16]. *S. aureus* is an important anthropological bacterial pathogen of Gram-positive strain that leads to common infections in society. Regarding the resistance to antibiotics, and the report of the Centers for Disease Control and Prevention (CDC) about resistance to methicillin of *S. aureus* (MRSA) in 2013, *S. aureus* was distinguished as a critical and a persistent threat [17–19].

**Figure 1:**
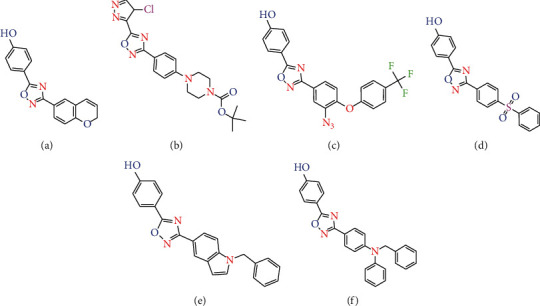
Examples of diverse structure for the 1,2,4-oxadiazole compounds.

The Gram-positive bacteria *S. aureus* is communal to humans and exists on the mucosa and skin of 30% of the population [20, 21]. It is a chief reason for hospital infections, the most common and serious of which are endocarditis and bacteremia endocarditis in hospitalized patients [22–25]. This organism has created resistance to a broad range of antibiotic medicine types [26]. Principal commercial compounds of the antimicrobial class (such as ciprofloxacin, ampicillin, and posaconazole) have limited performance against resistance microorganisms strains [27–30]. The erratic usage of antibiotics is known as one of the primary reasons for the increase in resistance of bacteria. The growth of bacterial resistance has resulted in a significant rise in mortality rates of individuals around the world [31]. On this path, there is a serious necessity to discover novel molecules with more effective antibacterial features, as well as obvious synthetic routes. This led to widespread research such as designing heterocyclic derivatives (like 1,2,4-oxadiazoles) with antimicrobial properties to treat *S. aureus* infections [32–36]. These discovered antibiotics are active and exhibit gram-positive activity, particularly against Staphylococcus aureus, including vancomycin-resistant, methicillin-resistant (MRSA), and linezolid-resistant by inhibiting srtA [24, 35, 37]. 1,2,4-Oxadiazole heterocycle was first manufactured in 1884. They showed remarkable action in vitro and in vivo and are orally bioavailable. The medicinal chemistry literatures report diverse structures for the 1,2,4-oxadiazoles (Figure 1) [38, 39]. In the present article, we represent 3D-QSAR investigations concerning 120 molecules of 1,2,4-oxadiazoles with antibacterial healing properties. This class of compounds (oxadiazoles) targets SrtA of the cell wall and inhibits it [40]. The advancement of antibiotics, especially of those that target cells of bacteria and have a desirable characteristic of toleration and safety, has largely helped population growth and has improved the quality of life in the last 75 years.

## 2. Materials and Methods

### 2.1. Data Set

A collection containing 120 compounds having 1,2,4-oxadiazole as antimicrobials was taken from the available literature [24] and was employed in the present study. All structures were extracted from Chembl (https://www.ebi.ac.uk/chembldb). The chosen compounds for the set of data shared a similar evaluation method with notable changes in their strength profiles and their structures. The compounds incorporated in the collection of datasets have antibiotics potencies with MIC values varying from 2 to 500 *μ*g/ml which were changed to M (molar). These were then converted to pMICs according to the following equation [41–44].

pMIC = −log_10_ [MIC].

**Figure 2:**
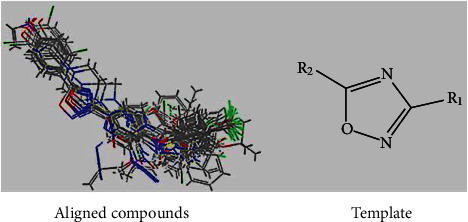
3D-QSAR structure superposition and alignment of the series (Strick model).

**Figure 3:**
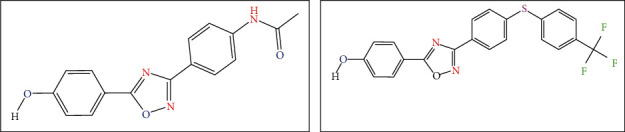
(b) Structure of compound **89** with the best active. (a) Structure of compound **120** with the lowest active.

The ligand 3D-formula of compounds was produced utilizing the Maestro v2015-2 and afterwards corrected using the LigPrep. Partial charges of atoms were attributed, and potential ionization was calculated at a neutral medium. The force field of OPLS_2005 was utilized for minimization to the conformer creation with the low energy of ligand. The minimization of energy was done for every compound (ligand) to reach an RMSD cutoff of 0.01 Å. Then, the final structures were used for modeling investigation. Additionally, all the 120 molecules were aligned in the method of alignment (Figure 2) relying on Maestro through choosing a common structure minimum as “template” and the most efficient one (compound **89**) as the “Reference Molecule” (Figure 3). Of the 120 molecules recognized in this investigation, a training group, including 96 molecules and a test group, including 24 molecules was created in Maestro [45–48].

### 2.2. Model Validation and Statistical Analysis

A high *q*^2^ only indicates a good internal validation in the training group, but it does not show a high prediction capability of the created models; hence, an external validation was necessary. The proved capability from generated models of 3D-QSAR was confirmed by computing the biological activities of compounds that applied as a test set and not inserted in the training set (Suppl. Table 1). In the present investigation, eighty percent of the molecules from the data set was accidentally chosen as training set models based on the atom field which were created of PLS factors (one until seven), and the obtained models were approved after predicting the activity of the test group ligand. The value of the model's prediction was assessed through the leave-one-out (LOO). The *q*^2^ (predictive correlation coefficient) was determined by utilizing Equation (1) [49].

(1) $$\begin{array}{c} q^{2}=1-\frac{\sum {\left(Y_{\text{predicted}}-Y_{\text{observed}}\right)}^{2}}{\sum {\left(Y_{\text{observed}}-Y_{\text{mean}}\right)}^{2}}. \end{array}$$

In the above equation, each of the three indices including *Y*_mean_, *Y*_predicted_, and *Y*_observed_, demonstrates the mean values predicted and observed of the pMIC feature, respectively. The  (*Y*_predicted_ − *Y*_observed_)^2^ index displays PRESS (the predictive residual sum of squares). The *r*_pred^2^ index related to the predictive correlation coefficient (*r*_pred^2^ > 0.6) [50] is calculated for the test group and is characterized through Equation (2).

(2) $$\begin{array}{c} r_{\text{pred}}^{2}=\left(\frac{\text{SD}-\text{PRESS}}{\text{SD}}\right) \end{array}$$

In Equation (2), the SD index shows the squared deviation sum for molecules amongst the test group biological activities and training group mean activities [51]. Also, the PRESS index indicates the squared deviation summation amongst actual and predicted activity values for molecules individually in the test group. Based on previous studies [52], If *R*^2^ is bigger than 0.6, and *R*^2^cv (*Q*^2^) is bigger than 0.5, 3D-QSAR models are acceptable. The regression model action composed was assessed using the RMSE index. For the data group, RMSE is computed as Equation (3) [53].

(3) $$\begin{array}{c} \text{RMSE}=\sqrt{\frac{\Sigma_{i=1}^{n}{\left(y_{i}-{\overset{\wedge }{y}}_{l}\right)}^{2}}{n}}. \end{array}$$

**Figure 4:**
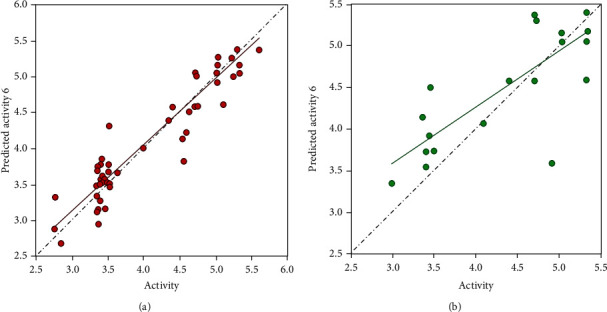
Scatter plot of the observed activity versus predicted activity of (a) training group compounds (*y* = 0.92*x* + 0.34, *R*^2^ = 0.92) and (b) test group compounds with the best fit line (*y* = 0.68*x* + 1.54, *R*^2^ = 0.65).

The 3D-QSAR model with the sixth component of the PLS factor was considered as the best for 1,2,4-oxadiazole derivatives. This model was approved for its precision in the ligand activity estimate in the training group [51]. Scatter plots for experimental and predicted activities of ligands showed a notable linear correlation. In Figures 4(a) and 4(b), the average difference of values of predicted and experimental for training and test groups is exposed, respectively.

### 2.3. Applicability Domain

APD can be determined using resemblance measures relying on the Euclidean distances between the entire compounds test and training. A comparison between the distance of the test compounds and their nearest neighbor to a predefined threshold in the training group is done, and the prediction is considered inaccurate when the interval is higher than that. The determination of APD was done based on the displayed formula, as follows.

(4) $$\begin{array}{c} \text{APD}=\left(d\right)+\text{Z}\delta . \end{array}$$

*δ* and *d* were calculated in a series of steps: first, the mean of Euclidean distances among all training compound pairs was estimated. Then, the collection of distances lower than the median was determined. *δ* and *d* were finally measured as the standard deviation and mean of distances that included in this set. The value equals 0.5 was selected for *Z*, which was the experimental cutoff in this study. For the applicability domain calculation, we utilized “AD using standardization approach” in DTC Lab (https://dtclab.webs.com/software-tools) [54–56].

*Y*-Randomization Test

The procedure of *Y*-randomization guarantees the validity of a 3D-QSAR model [57]. The dependent changeable vector is altered accidentally, and a novel 3D-QSAR model is produced. The strategy is repeated several times and if the recently produced 3D-QSAR models show low *R*^2^ and *Q*^2^ values, the accurateness of the original model is confirmed [58].

### 2.4. Docking Studies

**Figure 5:**
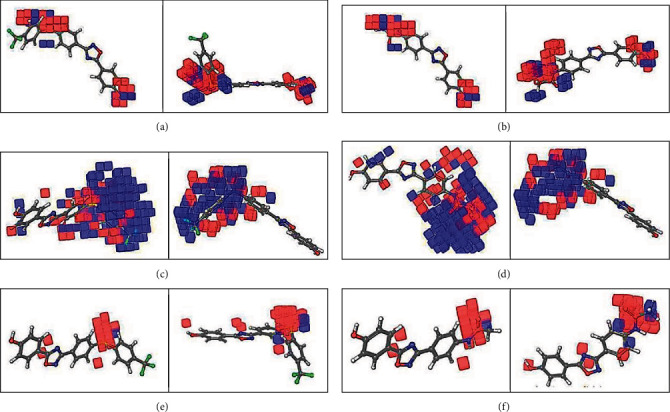
The visualizing of the 3D-QSAR model in the field of unfavorable and favorable effects of hydrogen bond donor (HBD) in: (a) ligand **89** and (b) ligand **120**. The visualizing of the 3D-QSAR model in the field of unfavorable and favorable effects of interaction in (c) ligand **89** and (d) ligand **120**. The visualizing of the 3D-QSAR model in the field of unfavorable and favorable effects of electron-withdrawing groups in (e) ligand **89** and (f) ligand **120** in two different dimensions, (positive coefficient color: dark blue, negative coefficient color: red, most active compound is **89** (pMIC = 5.617), and least active compound is **120** (pMIC = 2.771)).

**Figure 6:**
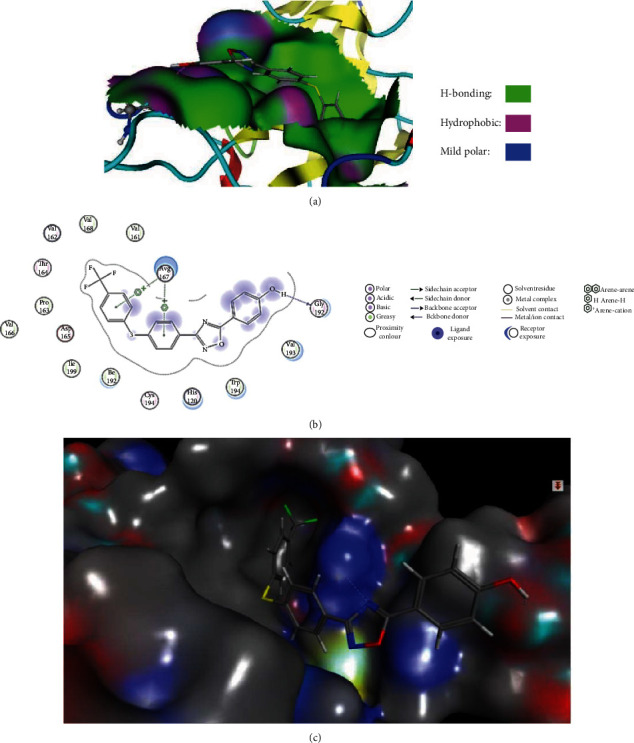
(a) The display of the image related to hydrophobic and hydrophilic fields for compound **89** into the active site (2KID). (b) The 2D pictures of the docked conformation of compound **89**. (c) The positioning of the compound **89** in the protein active site.

One most frequent tool for drug design is molecular docking, which employs a mode of association between binding sites of a suitable target with small molecules. Polypeptide structure, SrtA (accession number: 2KID), was acquired from the PDB data bank. Here, small-molecule docking in its active site and its analysis was done via Molecular Operation Environment (MOE) software (http://www.chemcomp.com) for selecting out the most active compound in terms of pMIC (pMIC = 2.77) with SrtA polypeptide. Before docking, the preparation of the ligand was done, and the 2D structure of ligand was set up by Chemoffice 12.0 which was further changed to 3D format by Hyper Chem7 software and was optimized employing PM3 semiempirical tool. Also, removal of crystallographic water molecules was done followed by association with pH 7 (for suitable ionization for both alkaline and acidic amino acids) and finally, hydrogen bonds were added. Utilizing the manual recommended parameters of the MOE energy minimization with a gradient of 0.05 and MMFF94X ff (force field), the energy of the retrieved protein molecule was calculated. The docking was done with force field as a filtration method via the triangle matcher placement, and the scoring function of the London DG algorithm in combination and the best conformation was analyzed in more details with the LigX module in MOE software. Docking was accomplished for the best compound (compound **89**) with the lowest MIC (Figure 5(a)) utilizing the default setting of MOE-Dock [12]. In the last section of the docking process, the selected ligand conformation was further investigated for its interactions of binding. The hydrophilic and hydrophobic field map for compound 89 was also formed (Figure 6(a)). The 2D pictures of the docked conformation of compound 89 are exhibited in Figure 6(b). The compound position in the protein active site is illustrated in Figure 6(c) [59–65].

## 3. Results and Discussion

### 3.1. 3D-QSAR Model

The 3D-QSAR model was created utilizing PLS regression statistics with the grid spacing 1 Å. The seven PLS factors were requested from the program, and the best model was observed for the sixth PLS factor owing to its high statistical importance and predictability. **(**Table 1**)** The fractions of field and parameters of statistical measured in QSAR-based Gaussian are organized in Table 2.

### 3.2. Model Validation

Validation of a common pharmacophore model and its prediction relying on active compounds were distinguished by *Q*_cv_^2^ = 0.5479 (Table 1). The training group *R*^2^ was 0.9235, which revealed the importance of this model. The produced model stability differs from 0.994 to 0.674. The value of *F* was observed to be 179.0. Moreover, a *P* value equal to 1.95*e*-047 and Pearson *r* equals 0.8050 showed an assurance of a higher degree in the model. The standard deviation and the root-mean-square error were equal to 0.2291 and 0.48, respectively, which depicts the strength of the created model in the test for the estimation of the unrecognized compounds. The values of measured pMIC related to the ligands which were included in the predicted group are summarized in Suppl. Table 1. *R*^2^ values greater than 0.5 as seen amongst the experimental and predicted values produced in the suitable model could show the inhibitory activity that was not included in the progression procedure. [66, 67]. These outcomes suggest that this method can analyze the QSAR model and the ligand-receptor interactions and could be employed in the design of new imidazole inhibitors. Scatter plots, given in Figures 3(a) and 3(b), showed a moderate distinction between the values of two groups, experimental and predicted, and striking linear correlation.

### 3.3. Applicability Domain

Reports of model constraints by the APD are critical. This shows an important aspect because the user can not only creatively and easily design new compounds but also they can be warned for the estimation validity as to when the structure features cannot be provided via the model. Therefore, after selecting the best model, the ADP of the model showed that the predicted model was valid. In the applicability domain, the compound was completely put inside the range. Indeed, all ligands were in the applicability domain and hence can be assumed as acceptable.

*Y*-Randomization Test

Further confirmation of the model was done via *Y*-randomization. Ten accidental changes of the *Y* vector were done, and the low values of *R*^2^ and *Q*^2^ were calculated. The range of the *R*^2^ and *Q*^2^ values were 0.34 to 0.57 and -0.45 to -0.65, respectively. It needs to be mentioned that every *Y* vector random stage was followed by the perfect training method to improve the new QSAR model, involving the choice of the most proper descriptors [68].

### 3.4. 3D-QSAR Contour Map Analysis

Contour plot interpretation was done to detect the influence of spatial arrangement on the structural characteristics like hydrophobic, ionic, electrostatic, H-bond acceptor, and H-bond donor locations on oxadiazole inhibitory effects. The positive contribution appeared in blue-colored cubes, and the negative contribution was visible in red. Figures 5(a)–5(f) are shown for the identification of the acceptable and unacceptable important interactions in two different dimensions, which resulted in the use of the QSAR model. HBD nature comparison of compound 89 (the best activity, Figure 5(a)) and the compound **120** (the least activity, Figure 5(b)) displays unacceptable and acceptable regions as red and blue cubes, respectively. Hydrogen bond donor maps showed that unfavorable locations placed next to the nitrogen atom of amide present on one side of the oxadiazole (Figure 5(a)) are an HBD group which is in the inappropriate place, whereas for the most active molecule, unfavorable regions lay near the sulfur atom which is not an HBD group (Figure 5(b)). Also, the hydrogen of the hydroxyl group on p-hydroxyphenyl, present on the other side of the oxadiazole near the desirable region, is available for two compounds—categorized as active and the least active.

Compounds such as ligand **89** with *p*-CF_3_-phenylthio hydrophobic substituent had higher potency values than compounds without substitute mentioned such as ligand **120**, because of the presence of favorable hydrophobic regions in that position (Figures 5(c) and 5(d)), which was confirmed by the results obtained from previous CoMFA studies [69]. For less active ligands such as compound **120**, the hydrophilic group (amid) fell into the favorable hydrophobic envelope that is not suitable for the hydrophilic groups. Comparison between the effects of the electron-withdrawing moieties of the best compound **89** with an electron p-CF3-phenylthio group and the least active compound **120** with the acetamido group was shown in Figures 5(f) and 5(e).

### 3.5. Docking Studies

The MOE-Dock program was utilized to check the stability of the models created in this study with the sortase A polypeptide receiver (PDB code 2KID). Studies of docking showed that interactions were commanded by aromaticity and hydrophobicity due to the position of phenol moiety (Figures 6(a)–6(c)). The best compound (pMIC = 5.617) was connected into the binding cavity of polypeptide SrtA with high affinity and created interactions in association with the oxygen of phenol with the Gly192 residue in one side of the ligand, while two rings on the other side of the oxadiazole have two interactions arene-cation with Arg 197 residue. The scores of docking studies of the best compound were -11.12 kcal/mol. Therefore, the compound **89** had a three-point attachment with the protein binding cavity. The interactions were present in the region containing Gly 192 and Arg 197 residues (Figure 6(b)). In general, oxygen is bound to hydrogen of hydroxyl in the acidic part of Gly 192 residue that showed only one hydrogen bond. This subject is visible by analyzing the hydrophilic and hydrophobic regions of compound 89 (Figure 6(a)). 3D-QSAR contour map analysis studies confirm this and showed that the compounds like ligand **89** are placed in a hydrophobic envelope (Figure 6(c)).

## 4. Conclusions

Using model prediction by 3D-QSAR studies of 120 analogs of 1,2,4-oxadiazoles and docking, we provided insights into the critical features needed for the design of inhibitors of SrtA. 3D-QSAR modeling was performed to provide a structural network for the comprehension of structure-activity relationships of the ligands present in the study. Studies of molecular docking were done to create desirable poses that bind to these compounds. The gets scores in VS (virtual screening) of compounds gave us chemically important points for the design and improvement of novel oxadiazoles as sortase inhibitors. The most active compound (**89)** of 1,2,4-oxadiazoles used in this study had four rings, named A, B, C, and D. HBD moieties in the A ring were essential for antibacterial activity. The aniline, phenol, and some heterocyclic compounds with hydrogen-bonding ability, such as pyrazoles, were allowed. These findings are in line with previous results. In line with previous explanations on the 3D-QSAR map analysis section, a hydrophobic substituent was seen essential for the activity in the D ring region [24, 69]. In general, by 3D-QSAR, we attempted to study the structural diversity in the ring D antibacterial activity in a 1,2,4-oxadiazoles region (Figure 1**)**. Finally, our findings suggest that the 1,2,4-oxadiazoles are inhibitors of sortase A and act against *S. aureus*, further holding great promising potential as future therapeutics for treating hospital infections.

## Figures and Tables

**Table 1:** PLS statistical parameters of the model QSAR model.

PLS		SD	*R* ^2^	*F*	*P*	Stability	RMSE	*Q* ^2^	Pearson-*R*
1		0.4285	0.7171	238.3	1.62*e*-027	0.994	0.45	0.6744	0.8302
2		0.3774	0.7830	167.8	1.41*e*-031	0.971	0.48	0.6323	0.8012
3		0.3254	0.8403	161.4	1.57*e*-036	0.907	0.44	0.6900	0.8342
4		0.2808	0.8824	170.7	2.1*e*-041	0.835	0.43	0.7056	0.8462
5		0.2503	0.9076	176.8	5.91*e*-045	0.773	0.45	0.6772	0.8314
6		0.2291	0.9235	179.0	1.95*e*-047	0.722	0.48	0.6319	0.8050
7		0.2070	0.9382	190.9	2.31*e*-050	0.674	0.49	0.6134	0.7970

SD: standard deviation of regression; *R*^2^: regression coefficient; *F*: variance ratio (ratio of the model variance to the observed activity variance); *P*: significance level of variance ratio; *Q*^2^: Crossvalidated correlation coefficient for the test group; RMSE: the RMS error in the test group predictions; Pearson-*R*: correlation among the predicted and observed activity for the test group.

**Table 2:** Seven factors of PLS were calculated for the QSAR model.

# factors	H-bond donor	Hydrophobic/nonpolar	Negative ionic	Positive ionic	Electron-withdrawing
1	0.028420	0.709533	0.030517	0.031581	0.156872
2	0.029319	0.646792	0.039072	0.039833	0.190441
3	0.028610	0.647774	0.045189	0.045139	0.200709
4	0.029662	0.668252	0.038533	0.037152	0.195964
5	0.029213	0.666539	0.039458	0.037827	0.195301
6	0.029931	0.669972	0.037575	0.035876	0.194004
7	0.032992	0.677689	0.033050	0.032073	0.191071

## Data Availability

The data about this original article are available upon reasonable request.

## Abbreviations

*R* ^2^: Regression coefficient
*Q* ^2^: Crossvalidation correlation coefficient
APD: Applicability domain
PLS: Partial least square
SD: Standard deviation
RMSD: Root mean square deviation
RMSE: Root mean squared error
HBD: Hydrogen-bond donor.

## Supplementary Materials

Supplementary Materials Supplementary Table 1: molecular view, assay-organism and assay-strain, the experimental, and predicted corresponding values of pMIC gained via QSAR models.
