# Supplementary material for: 3D-QSAR Studies of 1,2,4-Oxadiazole Derivatives as Sortase A Inhibitors
Source: Biomed Res Int. 2021 Dec 6;2021:6380336. doi: 10.1155/2021/6380336 (PMC8668286; doi:10.1155/2021/6380336)
Supplement: Supplementary Materials — Supplementary Table 1: molecular view, assay-organism and assay-strain, the experimental, and predicted corresponding values of pMIC gained via QSAR models. [file 6380336.f1.docx]

**Suppl Table 1:** Molecular view, assay-organism and assay-strain, the experimental, and predicted corresponding values of pMIC gained via QSAR models**.**

| Row | Chemical structure | **ASSAY_ORGANISM & ASSAY_STRAIN** | Experimental activity (pMIC) | Predicted activity (pMIC) | Residual activity |
| --- | --- | --- | --- | --- | --- |
| 1 | 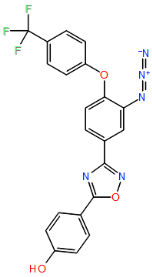 | **Antibacterial activity versus Staphylococcus epidermidis ATCC 35547** | 5.341781 | 5.15386 | -0.18792 |
| 2 | 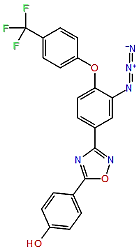 | **Antibacterial activity versus Vancomycin-susceptible Enterococcus faecium 119-39A** | 5.040751 | 4.9166 | -0.12415 |
| 3 | 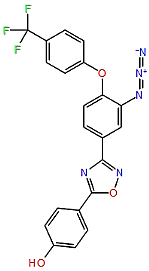 | **Antibacterial activity versus vancomycin-resistant Enterococcus faecalis 99** | 5.040751 | 5.15386 | 0.113109 |
| 4 | 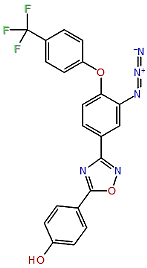 | **Antibacterial activity versus ciprofloxacin, gentamicin, oxacillin, penicillin, and linezolid-resistant Staphylococcus aureus NRS120 expressing mecA** | 5.040751 | 5.15386 | 0.113109 |
| 5 | 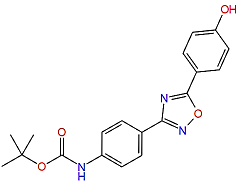 | **Antibacterial activity versus Staphylococcus aureus ATCC 29213** | 4.645152 | 4.50562 | -0.13953 |
| 6^t^ | 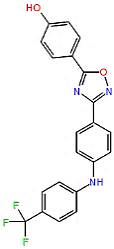 | **Antibacterial activity versus Staphylococcus aureus ATCC 29213** | 4.094034 | 4.07053 | -0.0235 |
| 7^t^ | 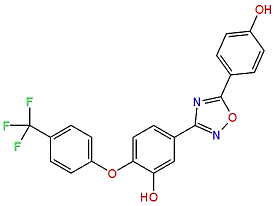 | **Antibacterial activity versus vancomycin-resistant Enterococcus faecium 106** | 4.714267 | 5.37159 | 0.657323 |
| 8^t^ | 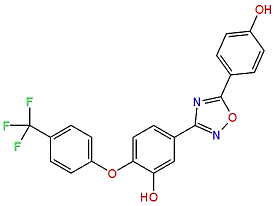 | **Antibacterial activity versus ciprofloxacin, gentamicin, oxacillin, penicillin, and linezolid-resistant Staphylococcus aureus NRS120 expressing mecA** | 4.714267 | 4.57655 | -0.13772 |
| 9 | 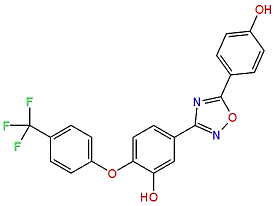 | **Antibacterial activity versus Enterococcus faecalis ATCC 29212** | 4.714267 | 4.57655 | -0.13772 |
| 10 | 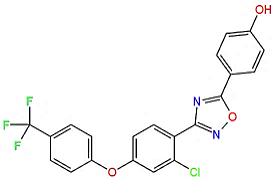 | **Antibacterial activity versus Bacillus licheniformis ATCC 12759** | 5.335247 | 5.04386 | -0.29139 |
| 11 | 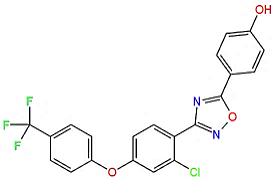 | **Antibacterial activity versus vancomycin-resistant Enterococcus faecium 106** | 5.034217 | 5.04389 | 0.009673 |
| 12 | 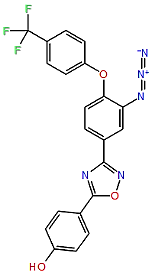 | **Antibacterial activity versus vancomycin-resistant Enterococcus faecium C68** | 5.040751 | 5.15386 | 0.113109 |
| 13 | 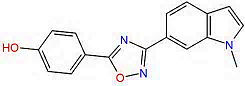 | **Antibacterial activity versus Staphylococcus aureus ATCC 29213** | 4.561265 | 4.13211 | -0.42916 |
| 14 | 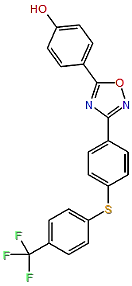 | **Antibacterial activity versus Enterococcus faecalis ATCC 29212** | 5.3164 | 5.37159 | 0.05519 |
| 15^t^ | 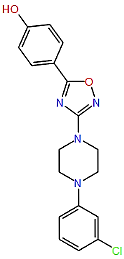 | **Antibacterial activity versus Staphylococcus aureus ATCC 29213** | 3.445227 | 3.91513 | 0.469903 |
| 16 | 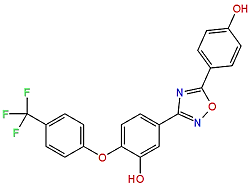 | **Antibacterial activity versus methicillin, oxacillin, and tetracycline-resistant and vancomycin and linezolid-susceptible Staphylococcus aureus ATCC 27660 expressing mecA** | 4.413237 | 4.57655 | 0.163313 |
| 17 | 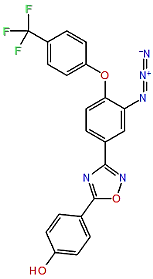 | **Antibacterial activity versus Enterococcus faecalis 201** | 5.040751 | 5.15386 | 0.113109 |
| 18 | 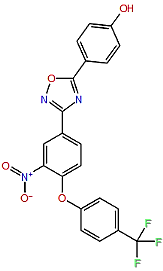 | **Antibacterial activity versus Staphylococcus aureus ATCC 29213** | 4.743647 | 5.00555 | 0.261903 |
| 19 | 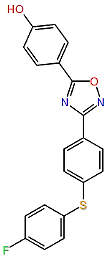 | **Antibacterial activity versus Staphylococcus aureus ATCC 29213** | 5.260548 | 4.99871 | -0.26184 |
| 20 | 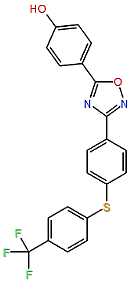 | **Antibacterial activity versus vancomycin-resistant Enterococcus faecalis 99** | 5.3164 | 5.37159 | 0.05519 |
| 21 | 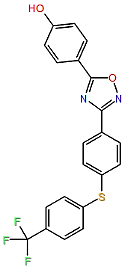 | **Antibacterial activity versus vancomycin-resistant Staphylococcus aureus VRS1** | 5.3164 | 5.37159 | 0.05519 |
| 22 | 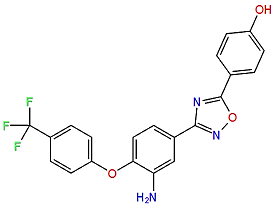 | **Antibacterial activity versus Staphylococcus aureus ATCC 29213** | 3.509118 | 3.77412 | 0.265002 |
| 23^t^ | 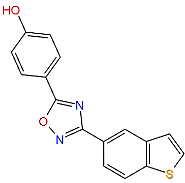 | **Antibacterial activity versus Staphylococcus aureus ATCC 29213** | 3.361625 | 4.13591 | 0.774285 |
| 24^t^ | 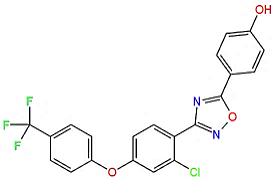 | **Antibacterial activity versus vancomycin-resistant Staphylococcus aureus VRS1** | 5.335247 | 5.04386 | -0.29139 |
| 25 | 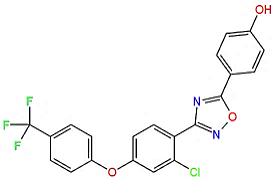 | **Antibacterial activity versus Vancomycin-susceptible Enterococcus faecium 119-39A** | 5.034217 | 5.04389 | 0.009673 |
| 26 | 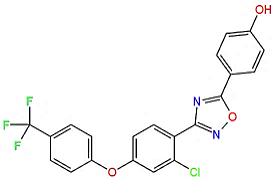 | **Antibacterial activity versus ciprofloxacin, gentamicin, oxacillin, penicillin, and linezolid-resistant Staphylococcus aureus NRS119 expressing mecA** | 5.034217 | 5.04389 | 0.009673 |
| 27 | 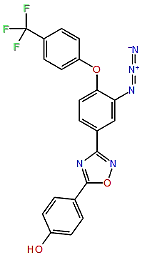 | **Antibacterial activity versus vancomycin-resistant Staphylococcus aureus VRS2** | 5.341781 | 5.15386 | -0.18792 |
| 28^t^ | 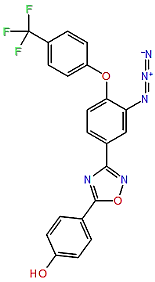 | **Antibacterial activity versus Enterococcus faecalis ATCC 29212** | 5.341781 | 5.15386 | -0.18792 |
| 29^t^ | 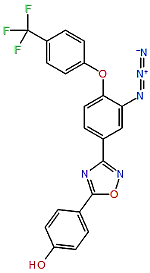 | **Antibacterial activity versus Bacillus licheniformis ATCC 12759** | 5.040751 | 5.15386 | 0.113109 |
| 30 | 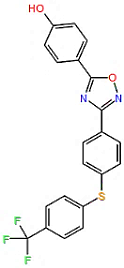 | **Antibacterial activity versus ciprofloxacin, gentamicin, oxacillin, penicillin, and linezolid-resistant Staphylococcus aureus NRS120 expressing mecA** | 5.3164 | 5.37159 | 0.05519 |
| 31 | 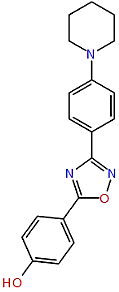 | **Antibacterial activity versus Staphylococcus aureus ATCC 29213** | 4.603929 | 4.22232 | -0.38161 |
| 32 | 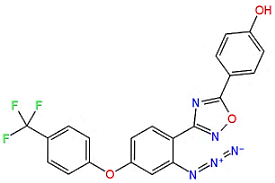 | **Antibacterial activity versus Staphylococcus aureus ATCC 29213** | 3.535601 | 3.46097 | -0.07463 |
| 33 | 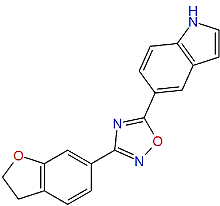 | **Antibacterial activity versus Staphylococcus aureus ATCC 29213** | 3.374691 | 2.93613 | -0.43856 |
| 34 | 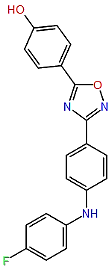 | **Antibacterial activity versus Staphylococcus aureus ATCC 29213** | 3.433557 | 3.52276 | 0.089203 |
| 35 | 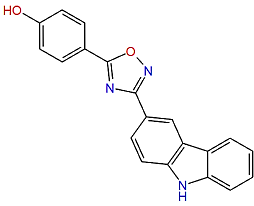 | **Antibacterial activity versus Staphylococcus aureus ATCC 29213** | 3.407789 | 3.56566 | 0.157871 |
| 36^t^ | 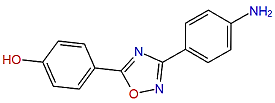 | **Antibacterial activity versus Staphylococcus aureus ATCC 29213** | 2.995327 | 3.34816 | 0.352833 |
| 37 | 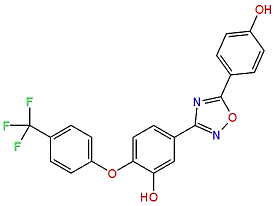 | **Antibacterial activity versus Vancomycin-susceptible Enterococcus faecium 119-39A** | 4.714267 | 4.57655 | -0.13772 |
| 38 | 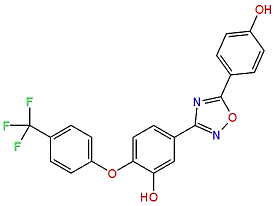 | **Antibacterial activity versus Bacillus licheniformis ATCC 12759** | 4.413237 | 4.57655 | 0.163313 |
| 39 | 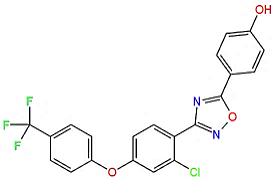 | **Antibacterial activity versus Staphylococcus haemolyticus ATCC 29970** | 5.034217 | 5.04389 | 0.009673 |
| 40^t^ | 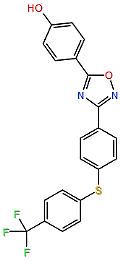 | **Antibacterial activity versus Staphylococcus epidermidis ATCC 35547** | 5.3164 | 5.37159 | 0.05519 |
| 41 | 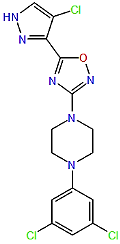 | **Antibacterial activity versus Staphylococcus aureus ATCC 29213** | 3.494492 | 3.5057 | 0.011208 |
| 42 | 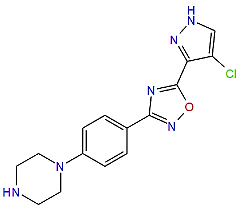 | **Antibacterial activity versus Staphylococcus aureus ATCC 29213** | 3.412329 | 3.7715 | 0.359171 |
| 43 | 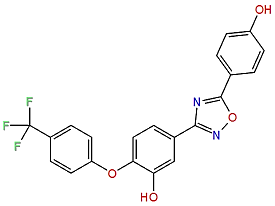 | **Antibacterial activity versus vancomycin-resistant Staphylococcus aureus VRS1** | 4.714267 | 4.57655 | -0.13772 |
| 44 | 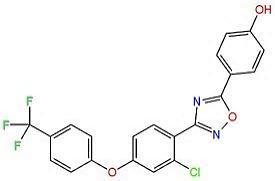 | **Antibacterial activity versus vancomycin-resistant Enterococcus faecalis 99** | 5.034217 | 5.04389 | 0.009673 |
| 45 | 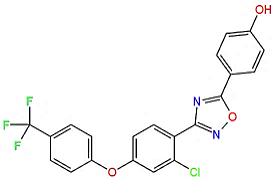 | **Antibacterial activity versus Enterococcus faecalis ATCC 29212** | 5.034217 | 5.04389 | 0.009673 |
| 46 | 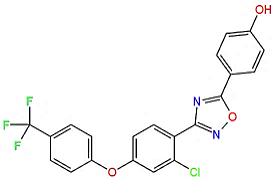 | **Antibacterial activity versus methicillin, oxacillin, and tetracycline-resistant and vancomycin and linezolid-susceptible Staphylococcus aureus NRS100 COL expressing mecA** | 4.733187 | 5.04389 | 0.310703 |
| 47 | 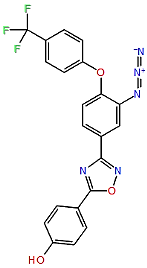 | **Antibacterial activity versus vancomycin-resistant Staphylococcus aureus VRS1** | 5.341781 | 5.15386 | -0.18792 |
| 48 | 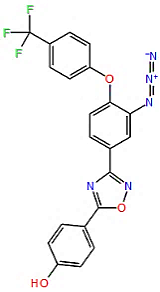 | **Antibacterial activity versus Bacillus cereus ATCC 13061** | 5.040751 | 5.15386 | 0.113109 |
| 49 | 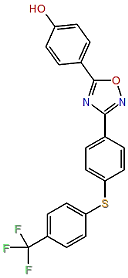 | **Antibacterial activity versus vancomycin-resistant Staphylococcus aureus VRS2** | 5.3164 | 5.37159 | 0.05519 |
| 50 | 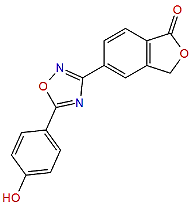 | **Antibacterial activity versus Staphylococcus aureus ATCC 29213** | 3.361536 | 3.32869 | -0.03285 |
| 51^t^ | 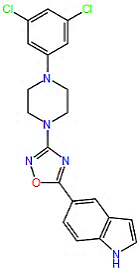 | **Antibacterial activity versus Staphylococcus aureus ATCC 29213** | 3.510105 | 3.72714 | 0.217035 |
| 52 | 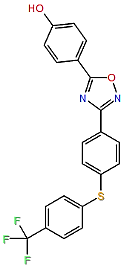 | **Antibacterial activity versus Vancomycin-susceptible Enterococcus faecium 119-39A** | 5.3164 | 5.37159 | 0.05519 |
| 53 | 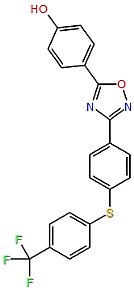 | **Antibacterial activity versus ciprofloxacin, gentamicin, oxacillin, penicillin, and linezolid-resistant Staphylococcus aureus NRS119 expressing mecA** | 5.3164 | 5.37159 | 0.05519 |
| 54^t^ | 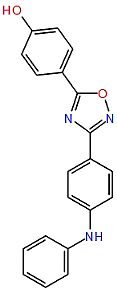 | **Antibacterial activity versus Staphylococcus aureus ATCC 29213** | 4.915611 | 3.5901 | -1.32551 |
| 55 | 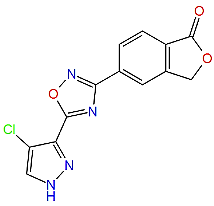 | **Antibacterial activity versus Staphylococcus aureus ATCC 29213** | 3.373774 | 3.47185 | 0.098076 |
| 56 | 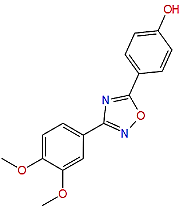 | **Antibacterial activity versus Staphylococcus aureus ATCC 29213** | 2.775683 | 3.31341 | 0.537727 |
| 57 | 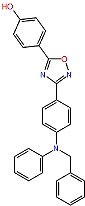 | **Antibacterial activity versus Staphylococcus aureus ATCC 29213** | 3.515501 | 3.66558 | 0.150079 |
| 58^t^ | 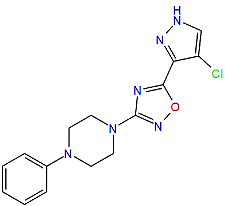 | **Antibacterial activity versus Staphylococcus aureus ATCC 29213** | 3.412329 | 3.7395 | 0.327171 |
| 59 | 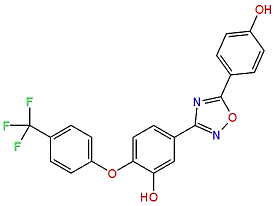 | **Antibacterial activity versus vancomycin-resistant Staphylococcus aureus VRS2** | 4.714267 | 4.57655 | -0.13772 |
| 60 | 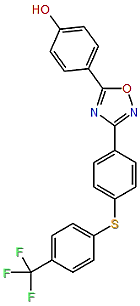 | **Antibacterial activity versus vancomycin-resistant Enterococcus faecium C68** | 5.3164 | 5.37159 | 0.05519 |
| 61 | 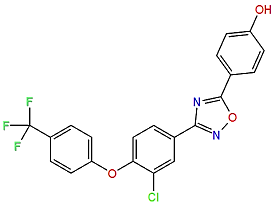 | **Antibacterial activity versus Staphylococcus aureus ATCC 29213** | 3.529067 | 4.30289 | 0.773823 |
| 62 | 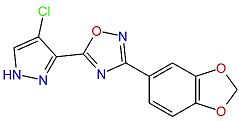 | **Antibacterial activity versus Staphylococcus aureus ATCC 29213** | 3.35619 | 3.10514 | -0.25105 |
| 63 | 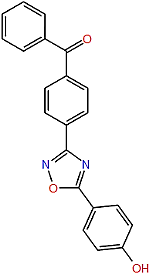 | **Antibacterial activity versus Staphylococcus aureus ATCC 29213** | 3.42726 | 3.62231 | 0.19505 |
| 64 | 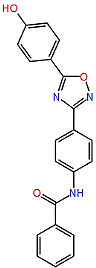 | **Antibacterial activity versus Staphylococcus aureus ATCC 29213** | 2.854148 | 2.66914 | -0.18501 |
| 65 | 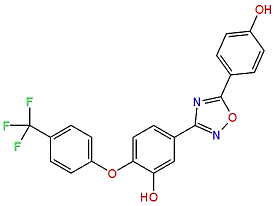 | **Antibacterial activity versus Enterococcus faecalis 201** | 4.714267 | 4.57655 | -0.13772 |
| 66^t^ | 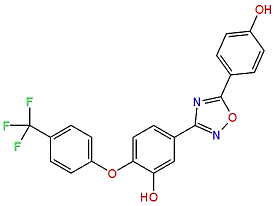 | **Antibacterial activity versus Staphylococcus haemolyticus ATCC 29970** | 4.714267 | 4.57655 | -0.13772 |
| 67 | 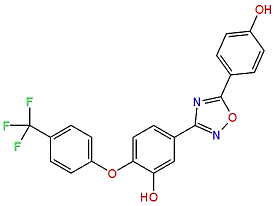 | **Antibacterial activity versus methicillin, oxacillin, and tetracycline-resistant and vancomycin and linezolid-susceptible Staphylococcus aureus NRS100 COL expressing mecA** | 4.413237 | 4.57655 | 0.163313 |
| 68 | 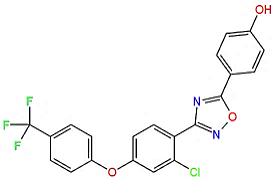 | **Antibacterial activity versus vancomycin-resistant Staphylococcus aureus VRS2** | 5.335247 | 5.04386 | -0.29139 |
| 69 | 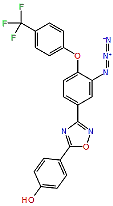 | **Antibacterial activity versus vancomycin-resistant Enterococcus faecium 106** | 5.040751 | 5.15386 | 0.113109 |
| 70 | 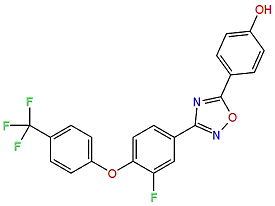 | **Antibacterial activity versus Staphylococcus aureus ATCC 29213** | 4.716348 | 4.57069 | -0.14566 |
| 71 | 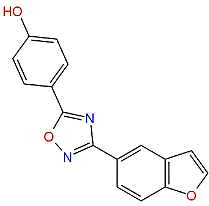 | **Antibacterial activity versus Staphylococcus aureus ATCC 29213** | 3.638286 | 3.65641 | 0.018124 |
| 72 | 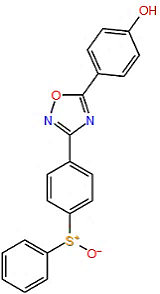 | **Antibacterial activity versus Staphylococcus aureus ATCC 29213** | 3.45199 | 3.59391 | 0.14192 |
| 73 | 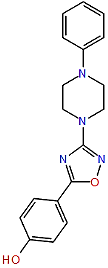 | **Antibacterial activity versus Staphylococcus aureus ATCC 29213** | 3.401145 | 3.26525 | -0.1359 |
| 74^t^ | 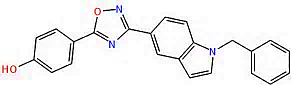 | **Antibacterial activity versus Staphylococcus aureus ATCC 29213** | 3.457941 | 4.49009 | 1.032149 |
| 75 | 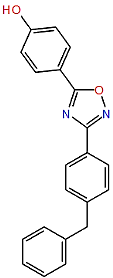 | **Antibacterial activity versus Staphylococcus aureus ATCC 29213** | 3.409154 | 3.50062 | 0.091466 |
| 76^t^ | 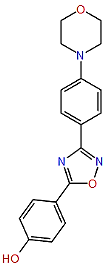 | **Antibacterial activity versus Staphylococcus aureus ATCC 29213** | 3.402463 | 3.54232 | 0.139857 |
| 77^t^ | 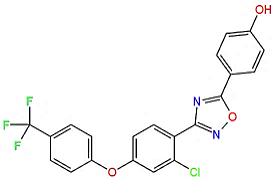 | **Antibacterial activity versus Bacillus cereus ATCC 13061** | 5.034217 | 5.04389 | 0.009673 |
| 78 | 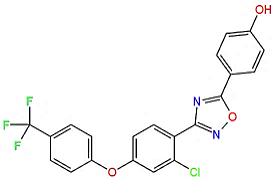 | **Antibacterial activity versus vancomycin-resistant Enterococcus faecium C68** | 5.034217 | 5.04389 | 0.009673 |
| 79 | 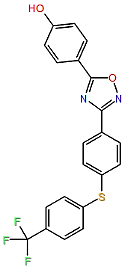 | **Antibacterial activity versus methicillin, oxacillin, and tetracycline-resistant and vancomycin and linezolid-susceptible Staphylococcus aureus ATCC 27660 expressing mecA** | 5.3164 | 5.37159 | 0.05519 |
| 80^t^ | 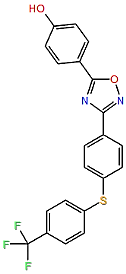 | **Antibacterial activity versus methicillin, oxacillin, and tetracycline-resistant and vancomycin and linezolid-susceptible Staphylococcus aureus NRS100 COL expressing mecA** | 5.3164 | 5.37159 | 0.05519 |
| 81 | 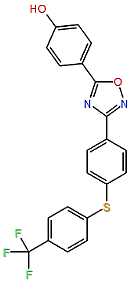 | **Antibacterial activity versus Bacillus cereus ATCC 13061** | 4.71434 | 4.57655 | -0.13779 |
| 82^t^ | 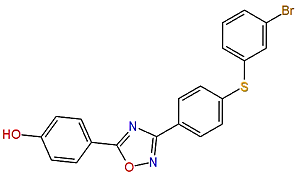 | **Antibacterial activity versus Staphylococcus aureus ATCC 29213** | 4.725616 | 5.30799 | 0.582374 |
| 83 | 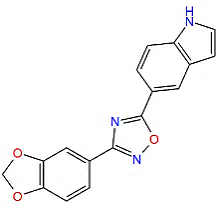 | **Antibacterial activity versus Staphylococcus aureus ATCC 29213** | 3.377503 | 3.14668 | -0.23082 |
| 84 | 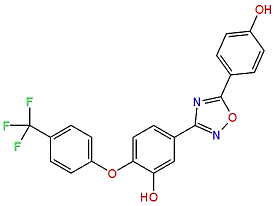 | **Antibacterial activity versus Bacillus cereus ATCC 13061** | 4.714267 | 4.57655 | -0.13772 |
| 85 | 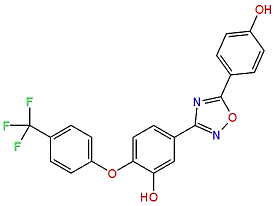 | **Antibacterial activity versus ciprofloxacin, gentamicin, oxacillin, penicillin, and linezolid-resistant Staphylococcus aureus NRS119 expressing mecA** | 4.714267 | 4.57655 | -0.13772 |
| 86 | 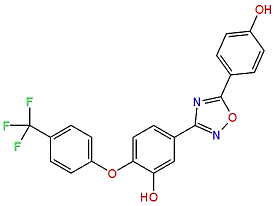 | **Antibacterial activity versus Staphylococcus epidermidis ATCC 35547** | 4.413237 | 4.57655 | 0.163313 |
| 87 | 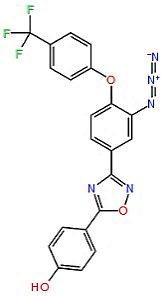 | **Antibacterial activity versus Staphylococcus haemolyticus ATCC 29970** | 5.341781 | 5.2049 | -0.13688 |
| 88 | 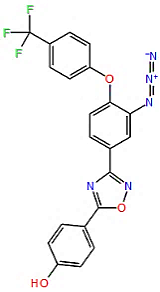 | **Antibacterial activity versus methicillin, oxacillin, and tetracycline-resistant and vancomycin and linezolid-susceptible Staphylococcus aureus ATCC 27660 expressing mecA** | 5.040751 | 5.15386 | 0.113109 |
| 89 | 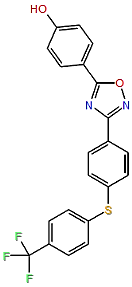 | **Antibacterial activity versus Bacillus licheniformis ATCC 12759** | 5.61743 | 5.37159 | -0.24584 |
| 90 | 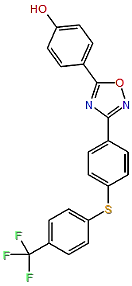 | **Antibacterial activity versus vancomycin-resistant Enterococcus faecium 106** | 5.3164 | 5.37159 | 0.05519 |
| 91 | 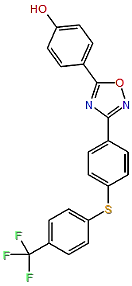 | **Antibacterial activity versus Staphylococcus aureus ATCC 29213** | 5.3164 | 5.37159 | 0.05519 |
| 92 | 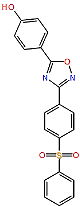 | **Antibacterial activity versus Staphylococcus aureus ATCC 29213** | 3.470753 | 3.15237 | -0.31838 |
| 93 | 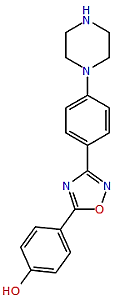 | **Antibacterial activity versus Staphylococcus aureus ATCC 29213** | 4.003205 | 3.99528 | -0.00793 |
| 94 | 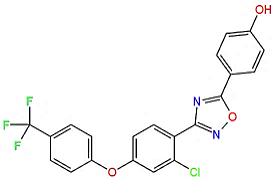 | **Antibacterial activity versus Staphylococcus aureus ATCC 29213** | 5.335247 | 5.04386 | -0.29139 |
| 95 | 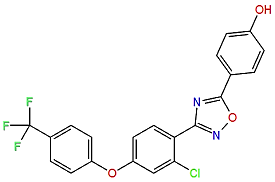 | **Antibacterial activity versus ciprofloxacin, gentamicin, oxacillin, penicillin, and linezolid-resistant Staphylococcus aureus NRS120 expressing mecA** | 5.034217 | 5.04389 | 0.009673 |
| 96 | 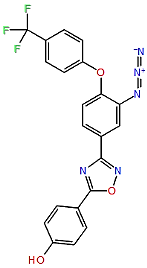 | **Antibacterial activity versus Staphylococcus aureus ATCC 29213** | 5.341781 | 5.15386 | -0.18792 |
| 97 | 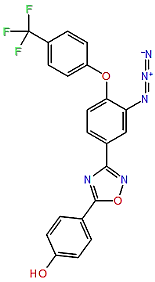 | **Antibacterial activity versus ciprofloxacin, gentamicin, oxacillin, penicillin, and linezolid-resistant Staphylococcus aureus NRS119 expressing mecA** | 5.040751 | 5.15386 | 0.113109 |
| 98^t^ | 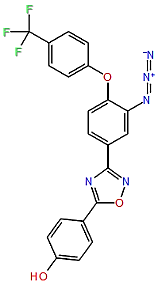 | **Antibacterial activity versus methicillin, oxacillin, and tetracycline-resistant and vancomycin and linezolid-susceptible Staphylococcus aureus NRS100 COL expressing mecA** | 5.040751 | 5.15386 | 0.113109 |
| 99 | 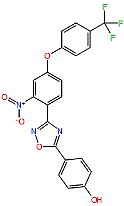 | **Antibacterial activity versus Staphylococcus aureus ATCC 29213** | 5.044677 | 5.26138 | 0.216703 |
| 100^t^ | 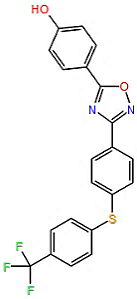 | **Antibacterial activity versus Staphylococcus haemolyticus ATCC 29970** | 5.3164 | 5.37159 | 0.05519 |
| 101 |  | **Antibacterial activity versus Staphylococcus aureus ATCC 29213** | 3.426015 | 3.84138 | 0.415365 |
| 102 |  | **Antibacterial activity versus Staphylococcus aureus ATCC 29213** | 4.358435 | 4.38963 | 0.031195 |
| 103 |  | **Antibacterial activity versus Staphylococcus aureus ATCC 29213** | 3.419646 | 3.56464 | 0.144994 |
| 104^t^ |  | **Antibacterial activity versus vancomycin-resistant Enterococcus faecalis 99** | 4.413237 | 4.57655 | 0.163313 |
| 105 |  | **Antibacterial activity versus Enterococcus faecalis 201** | 5.034217 | 5.04389 | 0.009673 |
| 106^t^ |  | **Antibacterial activity versus Staphylococcus epidermidis ATCC 35547** | 5.034217 | 5.04389 | 0.009673 |
| 107 |  | **Antibacterial activity versus Staphylococcus aureus ATCC 29213** | 4.561265 | 3.8144 | -0.74687 |
| 108 |  | **Antibacterial activity versus Staphylococcus aureus ATCC 29213** | 5.23856 | 5.25735 | 0.01879 |
| 109 |  | **Antibacterial activity versus Staphylococcus aureus ATCC 29213** | 3.343439 | 3.47885 | 0.135411 |
| 110 |  | **Antibacterial activity versus Staphylococcus aureus ATCC 29213** | 3.472402 | 3.53177 | 0.059368 |
| 111 |  | **Antibacterial activity versus Staphylococcus aureus ATCC 29213** | 3.527167 | 3.51316 | -0.01401 |
| 112 |  | **Antibacterial activity versus Staphylococcus aureus ATCC 29213** | 5.316327 | 5.37159 | 0.055263 |
| 113 |  | **Antibacterial activity versus Staphylococcus aureus ATCC 29213** | 4.754348 | 4.58456 | -0.16979 |
| 114^t^ |  | **Antibacterial activity versus Enterococcus faecalis 201** | 5.3164 | 4.57655 | -0.73985 |
| 115 |  | **Antibacterial activity versus vancomycin-resistant Enterococcus faecium C68** | 4.714267 | 4.57655 | -0.13772 |
| 116 |  | **Antibacterial activity versus methicillin, oxacillin, and tetracycline-resistant and vancomycin and linezolid-susceptible Staphylococcus aureus ATCC 27660 expressing mecA** | 4.733187 | 5.04389 | 0.310703 |
| 117 |  | **Antibacterial activity versus Staphylococcus aureus ATCC 29213** | 5.11747 | 4.60753 | -0.50994 |
| 118 |  | **Antibacterial activity versus Staphylococcus aureus ATCC 29213** | 3.369506 | 3.73756 | 0.368054 |
| 119 |  | **Antibacterial activity versus Staphylococcus aureus ATCC 29213** | 3.358604 | 3.68094 | 0.322336 |
| 120 |  | **Antibacterial activity versus Staphylococcus aureus ATCC 29213** | 2.771293 | 2.87339 | 0.102097 |

^t^: test
